# A Rare Interstitial Duplication of 8q22.1–8q24.3 Associated with Syndromic Bilateral Cleft Lip/Palate

**DOI:** 10.1155/2014/730375

**Published:** 2014-11-25

**Authors:** Regina Ferreira Rezek, Ana Angélica Rodrigues Abbas, Juliana Forte Mazzeu, Siliana Maria Duarte Miranda, Cibele Velloso-Rodrigues

**Affiliations:** ^1^Pontifical Catholic University of Minas Gerais (PUC Minas), 30535-901 Belo Horizonte, MG, Brazil; ^2^Catholic University of Brasília (UCB), 70790-160 Brasília, DF, Brazil; ^3^Genetic Technology Laboratory, BIOCOD, 30140-070 Belo Horizonte, MG, Brazil; ^4^Institute of Biological Science, Department of Basic Sciences, Genetics, Federal University of Juiz de Fora (UFJF), Campus Universitário, Rua José Lourenço Kelmer, s/n, Bairro São Pedro, 36036-900 Juiz de Fora, MG, Brazil

## Abstract

We present a rare case of 8q interstitial duplication derived from maternal balanced translocations in a patient with bilateral cleft lip and palate in syndromic form associated with other congenital malformations. G-banding cytogenetic analysis revealed a chromosomal abnormality in the form of the karyotype 46,XX der(22)t(8;22)(q22.1;p11.1)mat. Chromosome microarray analysis evidenced a 49 Mb duplicated segment of chromosome 8q with no pathogenic imbalances on chromosome 22. Two siblings also carry the balanced translocation. We have compared this case with other “pure” trisomies of 8q patients reported in the literature and with genome wide association studies recently published. This work highlights the involvement of chromosome 8q in orofacial clefts.

## 1. Introduction

Cleft lip and palate (CL/P) are the most common craniofacial human birth defects with an incidence of 1 in 800 births [1] with wide variability related to geographic origin [2] and socioeconomic status [3]. The prevalence of orofacial clefts in Brazil was 0.36 per 1,000 live births from 1998 to 2002 [4]. Their etiology involves genetic, epigenetic, and environmental factors. Some forms of orofacial clefts (OFCs) are associated with congenital malformations and chromosomal defects [5]. The classification of OFCs with or without other congenital anomalies, as well as chromosomal or gene variations, is described in the International Perinatal Database of Typical Oral Q1 Clefts [6] and it was modified in 2013 including new cleft subgroups [7].

In an analysis of medical records of 1127 patients, in the Cleft Palate/Craniofacial Clinic, Boys Town National Research Hospital, the authors found, among other findings, that 32.2% of all cleft patients had associated congenital malformations. Moreover, 63% of these patients with CL/P associated with congenital malformations had chromosome abnormalities [5]. At least ten chromosomes have been reported to be involved in the etiology of orofacial clefts (1p33; 1q32.2; 2p13; 2q33.1; 3q28; 3q28; 4p16.2; 4q21–q31; 6p24.3; 8q24.3; 11q23.3; 11q23.3; 13q33.1–q34; 14q22.2; and 19q13, MIM#119530). Some chromosomes are involved in the etiology of syndromic CL/P including trisomy 13 and trisomy 18 [8] and microdeletions 22q11.2 [9]. An extensive review about genetics of cleft lip and/or cleft palate based on publications identified by searching PubMed and OMIM has been published previously [10].

Pure distal 8q trisomy is a rare disorder. Phenotype varies in relation to the duplication size. Clinical signs include low birth weight; facial abnormalities such as prominent forehead, flat occiput, hypertelorism, upslanting palpebral fissure, nose and ear deformities, and thin upper lips; congenital heart defects; skeletal abnormalities; and retardation of growth and development [11–13].

This paper reports on a female patient, who exhibits a pattern of multiple congenital anomalies that includes CL/P and has a direct duplication (8) (8q22.1–8q24.3). This chromosomal anomaly resulted from a balanced translocation between chromosomes 8 and 22 in the mother. The balanced anomaly was inherited by two of the proband's male siblings.

## 2. Case Report

**Figure 1:**
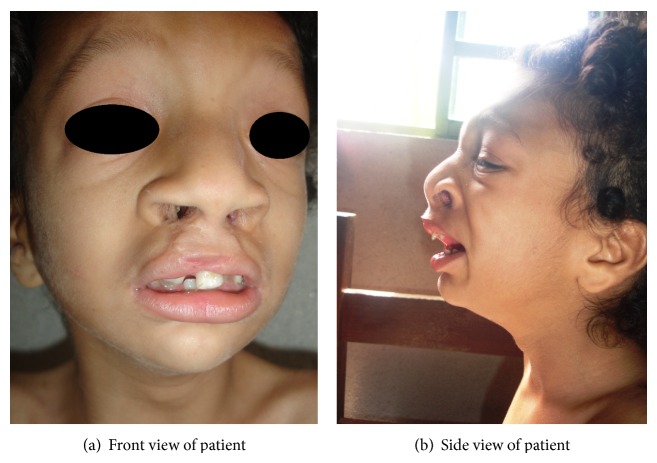
Patient at age of 8 years. Note craniofacial dysmorphism, bilateral transforaminal cleft (cheiloplasty and palatoplasty had been previously performed), high forehead, hypertelorism, low set ears, strabismus, and large nasal bridge with anteverted nares.

**Figure 2:**
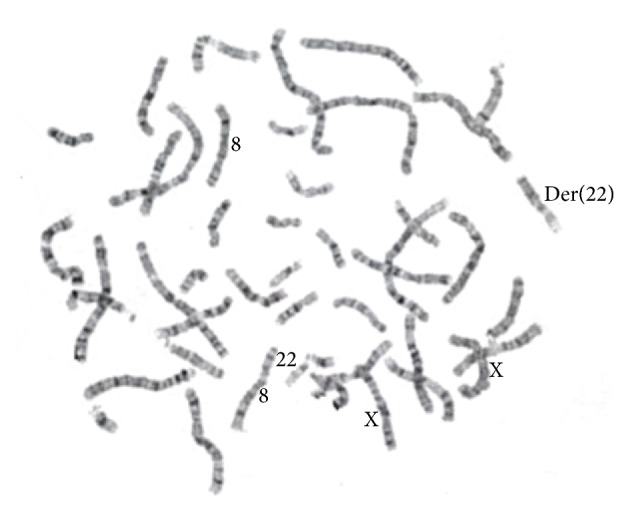
Micrograph of G-band metaphase chromosomes of the patient. Karyotype 46,XX der22t(8;22)(q22.1;p11.1)mat.

**Figure 3:**
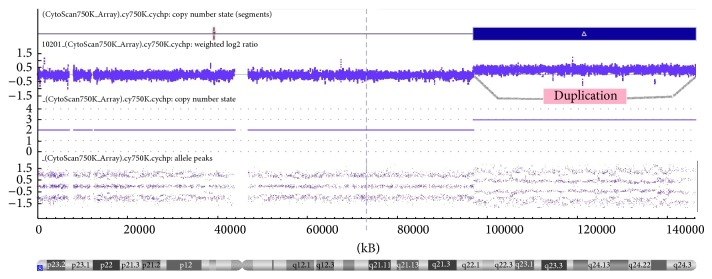
Ideogram showing duplication of 8q22.1–q24.3 detected by comparative genomic hybridization (CGH). Probe is ordered on the *x*-axis according to physical mapping positions, with the p-arm probes to the left and q-arm probes to the right. Highlighted region represents the duplication detected by microarray (chr8: 96,871,849–146,295,771, hg19).

The eight-year-old patient, Brazilian female, came to Center for Treatment and Rehabilitation of Cleft Lip/Palate and Craniofacial Deformities of the Pontifical Catholic University of Minas (CENTRARE/PUC Minas) for treatment of a bilateral transforaminal cleft; cheiloplasty and palatoplasty had been previously performed, but the patient presented with a new palatal opening. In addition to the CL/P, her clinical features comprise intellectual disability, speech and locomotion delay, frequent seizures, asthma, recurrent respiratory infections, worn teeth due to bruxism, high forehead, hypertelorism, low set ears, prominent nostrils, and a shallow nasal bridge, pectus excavatum, and long and thin fingers and toes (Figures 1(a) and 1(b)). Analysis of the family history identified that this female is the fourth of four children born to nonconsanguineous parents. Her three male siblings did not present phenotypic abnormalities. Chromosomal analysis by G-banding for the proband, the parents, and the siblings was requested. A 5 mL sample of peripheral blood was collected in a heparinized tube from the proband and her relatives. From this material, a temporary lymphocyte culture was performed to obtain cells in metaphase. Following cell culture with stimulation of chromosomal growth and elongation, G-band staining and cytogenetic microscopic analysis were performed to determine the constitutional alterations in the chromosomes. Thirty cells in metaphase were analyzed at a resolution of approximately 450 bands. As a reference, bands from the International System for Human Cytogenetic Nomenclature (ISCN, 2013) ideogram were used. Chromosomal microarray analysis was performed using CytoScan 750k plataform (Affymetrix, Inc.) according to the manufacturer's protocol. The proband had a derivative chromosome 22 of maternal origin. The translocation occurred between the short arm of chromosome 22 and the long arm of chromosome 8 (Figure 2). Subsequent analysis of parental chromosomes showed a balanced reciprocal translocation in the mother, with her karyotype being 46,XX,t(8;22)(p21.1;q22.3). Therefore, the patient had a partial trisomy of chromosome 8q with the karyotype 46,XX der(22)t(8;22)(q22.1;p11.1)mat. Chromosome microarray analysis evidenced a 49 Mb duplicated segment of chromosome 8 (chr8: 96,871,849–146,295,771, hg19) (Figure 3). No pathogenic imbalances were found on chromosome 22 or any other chromosomes. Two of the three phenotypically normal siblings carried the apparently balanced translocation.

## 3. Discussion

We report a patient with partial trisomy of chromosome 8 resulting from a balanced reciprocal translocation in the mother. The karyotype 46,XX der(22)t(8;22)(q22.1; p11.1)mat. appears to be responsible for the phenotype of the patient. Conventional karyotyping and molecular analysis of our patient revealed a duplication of approximately 49 Mb involving 8q22.1–8q24.3.

Within chromosome 8q, in the region corresponding to 49 Mb duplicated segment (8q22.1–8q24.3), there are 54 loci (OMIM, 2014), whose products, in triplicate, may have contributed to proband's phenotype. The region 8q24.3 was strongly associated with nonsyndromic cleft lip with or without cleft palate (NSCL/P) (MIM#612858) in genome wide association studies (GWAS) in patients of European descent [14, 15], in Mesoamerican [16] and Brazilian population [17] but not in the Japanese population [18], or in the southern Han Chinese population from Guangdong Province [19]. Fine mapping of the 640 kb region in 462 unrelated NSCL/P cases revealed a single nucleotide polymorphism (SNP) (rs987525) that was significantly associated with the disorder [14]. A meta-analysis for NSCL/P using data from the two largest GWA studies showed that the highest risk was associated with the A allele of SNP rs987525 on chromosome 8q24 at genomic position 8: 139,900,000–146,364,022 [20]. The locus has been named* OFC12* (orofacial cleft 12) but its function is still unknown.

Clinical signs of trisomy 8q23-qter include short stature, microcephaly, peculiar face, microphthalmia, cleft lip/palate, short and broad neck, widely spaced nipples, congenital heart defects, overriding toes, and mostly severe mental retardation [21]. Whilst some cases of 8q duplication have been reported, “pure” trisomy of 8q is rare and only a few patients showed OFC. The duplication in our patient overlaps with others that have been reported in the literature with a common region of overlap. Although there is a large overlap between regions (chr8: 45 Mb), two cases of interstitial duplication at 8q22.2–q24.3 (chr8: 100,338,614–145,464,363-hg18) [21] and at 8q23.3–q24.1 (116,147,673–129,419,458) [22] were reported in children with similar dysmorphic features but without OFC. Thus our proband has a large duplication of chromosome 8q that extends to the* OFC12* locus which explains the presence of OFC.

## 4. Conclusion

In conclusion, our case contributes to the definition of the critical region for etiology of OFC on distal chromosome 8q24.3 and provides new information to patients with this rare duplication syndrome.
